# Supplementary material for: HDAC Inhibitors Can Enhance Radiosensitivity of Head and Neck Cancer Cells Through Suppressing DNA Repair
Source: Cancers (Basel). 2024 Dec 7;16(23):4108. doi: 10.3390/cancers16234108 (PMC11640432; doi:10.3390/cancers16234108)
Supplement: Supplementary file 1 [file cancers-16-04108-s001.zip › cancers-3328655-supplementary.pdf]

Supplementary Table S1. List of drugs used in the spheroid screen

Code	Drug	Target
S1117	Triciribine	AKT
S7492	Uprosertib (GSK2141795)	AKT
S7521	Afuresertib (GSK2110183)	AKT
S1048	Tozasertib (VX-680, MK-0457)	Aurora Kinase
S1133	Alisertib (MLN8237)	Aurora Kinase
S1147	Barasertib (AZD1152-HQPA)	Aurora Kinase
S8048	Venetoclax (ABT-199, GDC-0199)	Bcl-2
S2248	Silmitasertib (CX-4945)	Casein Kinase
S7326	Tasisulam	Caspase
S7775	Emricasan	Caspase
S1153	Roscovitine (Seliciclib, CYC202)	CDK
S2768	Dinaciclib (SCH727965)	CDK
S7158	Abemaciclib (LY2835219)	CDK
S7440	Ribociclib (LEE011)	CDK
S1230	Flavopiridol (Alvocidib)	CDK
S1774	Thioguanine	DNA Methyltransferase
S1782	Azacitidine	DNA Methyltransferase
S1200	Decitabine	DNA Methyltransferase
S4294	Procainamide HCl	DNA Methyltransferase, Sodium Channel
S1156	Capecitabine	DNA/RNA Synthesis
S1192	Raltitrexed	DNA/RNA Synthesis
S1199	Cladribine	DNA/RNA Synthesis
S1209	Fluorouracil (5-Fluoracil, 5-FU)	DNA/RNA Synthesis
S1212	Bendamustine HCl	DNA/RNA Synthesis
S1213	Nelarabine	DNA/RNA Synthesis
S1218	Clofarabine	DNA/RNA Synthesis
S1221	Dacarbazine	DNA/RNA Synthesis
S1229	Fludarabine Phosphate	DNA/RNA Synthesis
S1289	Carmofur	DNA/RNA Synthesis
S1299	Floxuridine	DNA/RNA Synthesis
S1300	Tegafur (FT-207, NSC 148958)	DNA/RNA Synthesis
S1302	Ifosfamide	DNA/RNA Synthesis
S1305	Mercaptopurine (6-MP)	DNA/RNA Synthesis
S1840	Lomustine	DNA/RNA Synthesis
S1896	Hydroxyurea	DNA/RNA Synthesis
S4252	Mechlorethamine HCl	DNA/RNA Synthesis
S4288	Chloroambucil	DNA/RNA Synthesis
S4504	6-MP Monohydrate	DNA/RNA Synthesis
S3669	Carmustine	DNA/RNA Synthesis
S5293	Nimustine Hydrochloride	DNA/RNA Synthesis
S5297	Vidarabine monohydrate	DNA/RNA Synthesis
S5582	Cytarabine hydrochloride	DNA/RNA Synthesis
S5552	Amenamevir	DNA/RNA Synthesis
S1760	Rifapentine	DNA/RNA Synthesis
S1764	Rifampin	DNA/RNA Synthesis
S1778	Trifluridine	DNA/RNA Synthesis
S1784	Vidarabine	DNA/RNA Synthesis
S1790	Rifaximin	DNA/RNA Synthesis
S1907	Metronidazole	DNA/RNA Synthesis
S2029	Uridine	DNA/RNA Synthesis
S2794	Sofosbuvir (PSI-7977, GS-7977)	DNA/RNA Synthesis
S3001	Clevudine	DNA/RNA Synthesis

S4227	Fidaxomicin	DNA/RNA Synthesis
S4297	Mupirocin	DNA/RNA Synthesis
S7975	Favipiravir (T-705)	DNA/RNA Synthesis
S8146	Mitomycin C	DNA/RNA Synthesis
S1334	Flupirtine maleate	DNA/RNA Synthesis
S1983	Adenine HCl	DNA/RNA Synthesis
S4035	Vitamin D2	DNA/RNA Synthesis
S5484	Rimantadine Hydrochloride	DNA/RNA Synthesis
S1214	Bleomycin sulphate	DNA/RNA Synthesis
S1237	Temozolomide	DNA/RNA Synthesis, Autophagy
S1949	Menadione	DNA/RNA Synthesis, phosphatase
S1491	Fludarabine	DNA/RNA Synthesis, STAT
S1025	Gefitinib (ZD1839)	EGFR
S1392	Pelitinib (EKB-569)	EGFR
S2727	Dacomitinib (PF299804, PF299)	EGFR
S2755	Varlitinib	EGFR
S2922	Icotinib	EGFR
S7284	Rociletinib (CO-1686, AVL-301)	EGFR
S7297	Osimertinib (AZD9291)	EGFR
S7786	Erlotinib	EGFR
S4667	Lidocaine hydrochloride	EGFR
S5098	Gefitinib hydrochloride	EGFR
S8294	Olmutinib (HM61713, BI 1482694)	EGFR, BTK
S1194	CUDC-101	EGFR, HDAC, HER2
S1028	Lapatinib (GW-572016) Ditosylate	EGFR, HER2
S7810	Afatinib (BIBW2992) Dimaleate	EGFR, HER2
S1011	Afatinib (BIBW2992)	EGFR, HER2
S2111	Lapatinib	EGFR, HER2
S1342	Genistein	EGFR, Topoisomerase
S7854	Ulixertinib (BVD-523, VRT752271)	ERK
S2823	Tideglusib	GSK-3
S1053	Entinostat (MS-275)	HDAC
S1085	Belinostat (PXD101)	HDAC
S1090	Abexinostat (PCI-24781)	HDAC
S1096	Quisinostat (JNJ-26481585) 2HCl	HDAC
S1122	Mocetinostat (MGCD0103)	HDAC
S1515	Pracinostat (SB939)	HDAC
S2693	Resminostat	HDAC
S4125	Sodium Phenylbutyrate	HDAC
S8001	Ricolinostat (ACY-1215)	HDAC
S1703	Divalproex Sodium	HDAC
S3944	Valproic acid	HDAC
S1030	Panobinostat (LBH589)	HDAC
S2216	Mubritinib (TAK 165)	HER2
S7358	Poziotinib (HM781-36B)	HER2, EGFR
S5500	Amodiaquine hydrochloride	HnMT
S7015	Birinapant	IAP
S7205	Idasanutlin (RG-7388)	Mdm2
S1008	Selumetinib (AZD6244)	MEK
S1475	Pimasertib (AS-703026)	MEK
S2673	Trametinib (GSK1120212)	MEK
S7007	Binimetinib (MEK162, ARRY-162, ARRY-438162)	MEK
S8041	Cobimetinib (GDC-0973, RG7420)	MEK
S7505	(S)-crizotinib	MTH1
S1044	Temsirolimus (CCI-779, NSC 683864)	mTOR

S1120	Everolimus (RAD001)	mTOR
S5003	Tacrolimus (FK506)	mTOR
S2658	Omipalisib (GSK2126458, GSK458)	mTOR, PI3K
S7646	Voxtalisib (XL765, SAR245409)	mTOR, PI3K
S5733	Stearic acid	NF-kB
S3137	Sodium salicylate	NF-κB
S4073	Sodium 4-Aminosalicylate	NF-κB
S1848	Curcumin	NF-κB, HDAC, HAT, Nrf2
S1574	Doramapimod (BIRB 796)	p38 MAPK
S7215	Losmapimod (GW856553X)	p38 MAPK
S7799	Pexmetinib (ARRY-614)	p38 MAPK, Tie-2
S1004	Veliparib (ABT-888)	PARP
S1060	Olaparib (AZD2281, Ku-0059436)	PARP
S1087	Iniparib (BSI-201)	PARP
S1098	Rucaparib (AG-014699, PF-01367338) phosphate	PARP
S7625	Niraparib (MK-4827) Tosylate	PARP
S2741	Niraparib (MK-4827)	PARP
S1470	Orantinib (TSU-68, SU6668)	PDGFR
S2730	Crenolanib (CP-868596)	PDGFR
S4736	Trapidil	PDGFR
S2475	Imatinib (STI571)	PDGFR
S7781	Sunitinib	PDGFR, c-Kit, VEGFR
S1040	Sorafenib Tosylate	PDGFR, Raf, VEGFR
S1065	Pictilisib (GDC-0941)	PI3K
S2226	Idelalisib (CAL-101, GS-1101)	PI3K
S2814	Alpelisib (BYL719)	PI3K
S7028	Duvelisib (IPI-145, INK1197)	PI3K
S7645	Pilaralisib (XL147)	PI3K
S1362	Rigosertib (ON-01910)	PLK
S2235	Volasertib (BI 6727)	PLK
S1729	Gemfibrozil	PPAR
S1794	Fenofibrate	PPAR
S2075	Rosiglitazone HCl	PPAR
S4527	Fenofibric acid	PPAR
S2505	Rosiglitazone maleate	PPAR
S2665	Ciprofibrate	PPAR
S4159	Bezafibrate	PPAR
S4207	Clofibric Acid	PPAR
S8432	Troglitazone (CS-045)	PPAR
S2590	Pioglitazone	PPAR
S2556	Rosiglitazone	PPAR
S5487	Cefoperazone sodium	PPARd
S1267	Vemurafenib (PLX4032, RG7204)	Raf
S2807	Dabrafenib (GSK2118436)	Raf
S5069	Dabrafenib Mesylate	Raf
S7108	Encorafenib (LGX818)	Raf
S7397	Sorafenib	Raf
S1130	Sepantronium Bromide (YM155)	Survivin
S4238	Cepharanthine	TNF-alpha
S1567	Pomalidomide	TNF-alpha
S1029	Lenalidomide (CC-5013)	TNF-alpha
S8034	Apremilast (CC-10004)	TNF-alpha, PDE
S1623	Acetylcysteine	TNF-alpha, ROS
S1208	Doxorubicin (Adriamycin) HCl	Topoisomerase
S1223	Epirubicin HCl	Topoisomerase
S1225	Etoposide	Topoisomerase
S1231	Topotecan HCl	Topoisomerase

S1367	Amonafide	Topoisomerase
S1787	Teniposide	Topoisomerase
S3035	Daunorubicin HCl	Topoisomerase
S1222	Dexrazoxane HCl (ICRF-187, ADR-529)	Topoisomerase
S2485	Mitoxantrone 2HCl	Topoisomerase
S2492	Novobiocin Sodium	Topoisomerase
S1340	Gatifloxacin	Topoisomerase
S1465	Moxifloxacin HCl	Topoisomerase
S1756	Enoxacin	Topoisomerase
S2064	Balofloxacin	Topoisomerase
S2328	Nalidixic acid	Topoisomerase
S3181	Flumequine	Topoisomerase
S4119	Pefloxacin Mesylate Dihydrate	Topoisomerase
S4591	Nitroxoline	Topoisomerase
S4604	Levofloxacin hydrate	Topoisomerase
S2217	Irinotecan HCl Trihydrate	Topoisomerase
S1228	Idarubicin HCl	Topoisomerase
S1940	Levofloxacin	Topoisomerase
S5059	Pixantrone Maleate	Topoisomerase

Supplementary Table S2. Relative FaDu spheroid growth following drug and radiation combinations

Drug	1 µM + IR	0.03 µM + IR
Pefloxacin Mesylate Dihydrate	441.7	221.9
Tacrolimus (FK506)	432.7	226.0
Everolimus (RAD001)	352.1	131.6
Mitoxantrone 2HCl	339.1	70.4
Ricolinostat (ACY-1215)	332.9	73.2
Idelalisib (CAL-101, GS-1101)	309.6	291.8
Sodium salicylate	289.9	129.8
Sodium 4-Aminosalicylate	284.6	219.2
Nitroxoline	275.9	169.8
Daunorubicin HCl	275.0	60.0
Ulixertinib (BVD-523, VRT752271)	265.1	87.9
Dexrazoxane HCl (ICRF-187, ADR-529)	253.4	44.8
Lapatinib (GW-572016) Ditosylate	251.8	49.5
Varlitinib	250.7	149.0
Temozolomide	248.3	248.3
Iniparib (BSI-201)	223.5	167.5
Voxtalisib (XL765, SAR245409)	222.5	131.3
Sorafenib Tosylate	216.1	192.9
Cepharanthine	215.4	213.9
Losmapimod (GW856553X)	215.2	183.9
Fluorouracil (5-Fluoracil, 5-FU)	208.5	101.1
Carmustine	207.3	366.2
6-Mercaptopurine (6-MP) Monohydrate	205.9	165.7
Nelarabine	203.1	89.0
Novobiocin Sodium	195.1	45.1
Apremilast (CC-10004)	194.2	48.2
Nimustine Hydrochloride	191.9	244.9
Lenalidomide (CC-5013)	190.4	74.3
Doramapimod (BIRB 796)	188.0	184.7
Idasanutlin (RG-7388)	185.7	174.6
Amonafide	182.3	69.0
Acetylcysteine	177.1	153.2
Doxorubicin (Adriamycin) HCl	173.0	167.8
Chloroambucil	169.4	259.0
Gatifloxacin	168.2	88.0
Gefitinib (ZD1839)	165.7	106.7
Roscovitine (Seliciclib,CYC202)	162.4	65.3
Rifapentine	160.9	146.7
Moxifloxacin HCl	160.5	207.8
Procainamide HCl	155.4	99.1
Entinostat (MS-275)	154.3	121.5
Duvelisib (IPI-145, INK1197)	151.6	0.0
Belinostat (PXD101)	150.9	103.5
Pictilisib (GDC-0941)	149.7	182.0
Veliparib (ABT-888)	147.8	187.7
Azacitidine	147.6	96.4
Balofloxacin	146.4	144.5
Etoposide	145.6	185.8
Venetoclax (ABT-199, GDC-0199)	144.8	106.2
Decitabine	144.0	144.8
Topotecan HCl	143.5	63.6
Olaparib (AZD2281, Ku-0059436)	141.0	90.2
Mechlorethamine HCl	138.0	175.2
Sunitinib	137.8	442.0

Pomalidomide	136.5	57.2
Rimantadine Hydrochloride	134.7	238.9
Metronidazole	134.3	147.4
Afatinib (BIBW2992)	132.8	29.1
Pilaralisib (XL147)	131.8	149.6
Valproic acid	128.0	124.2
Afatinib (BIBW2992) Dimaleate	127.6	75.1
Orantinib (TSU-68, SU6668)	125.3	44.9
Ribociclib (LEE011)	125.2	73.8
Uprosertib (GSK2141795)	123.5	161.7
Teniposide	122.7	65.2
Afuresertib (GSK2110183)	122.7	161.7
Favipiravir (T-705)	121.8	99.7
Thioguanine	119.3	104.9
Uridine	118.4	161.0
Vitamin D2	117.5	140.1
Divalproex Sodium	117.3	129.3
Bleomycin sulfate	116.6	97.6
Amenamevir	113.2	121.1
Amodiaquine hydrochloride	112.8	164.0
Trifluridine	111.9	105.3
Epirubicin HCl	110.9	147.2
Rifaximin	108.5	117.9
Mubritinib (TAK 165)	105.5	80.2
Tozasertib (VX-680, MK-0457)	104.9	98.1
Cytarabine hydrochloride	104.6	207.0
Capecitabine	103.1	127.7
Pexmetinib (ARRY-614)	101.0	76.0
Sofosbuvir (PSI-7977, GS-7977)	100.8	111.6
Enoxacin	97.9	29.4
Vidarabine	97.7	99.2
Silmitasertib (CX-4945)	97.0	106.0
Cladribine	94.5	79.4
Menadione	93.1	187.6
Triciribine	91.1	110.6
Lapatinib	90.0	140.0
Rifampin	89.3	104.5
Resminostat	87.6	36.8
Adenine HCl	86.2	83.8
Osimertinib (AZD9291)	85.3	163.3
Icotinib	85.1	42.6
Trapidil	83.6	136.0
Alisertib (MLN8237)	83.2	135.8
Nalidixic acid	81.6	120.3
Carmofur	80.5	48.8
Hydroxyurea	80.3	73.3
Lomustine	78.9	92.1
Dacarbazine	77.7	80.5
Alpelisib (BYL719)	76.4	151.3
Flupirtine maleate	75.3	85.6
Barasertib (AZD1152-HQPA)	75.1	87.7
Imatinib (STI571)	73.7	68.8
Volasertib (BI 6727)	72.5	170.0
Temsirolimus (CCI-779, NSC 683864)	71.3	125.4
Sodium Phenylbutyrate	70.6	152.7
Tegafur (FT-207, NSC 148958)	69.5	54.3
Clofarabine	68.4	62.7

Tideglusib	68.4	70.5
Fludarabine Phosphate	67.3	66.9
Birinapant	66.9	107.0
Vidarabine monohydrate	66.8	82.8
Clevudine	66.8	107.7
Tasisulam	65.9	123.8
Emricasan	63.8	79.9
Pelitinib (EKB-569)	63.6	133.7
Dacomitinib (PF299804, PF299)	63.6	133.7
Lidocaine hydrochloride	63.2	17.6
Crenolanib (CP-868596)	60.9	164.8
Abemaciclib (LY2835219)	60.6	85.7
Curcumin	59.7	76.3
Fludarabine	59.6	98.8
Gefitinib hydrochloride	58.9	8.2
Bendamustine HCl	58.2	47.2
Floxuridine	57.7	43.0
Mercaptopurine (6-MP)	56.6	51.4
Abexinostat (PCI-24781)	55.7	102.5
(S)-crizotinib	55.4	129.6
Mupirocin	54.7	43.3
Rosiglitazone HCl	53.9	11.0
Flumequine	53.8	177.8
Erlotinib	50.7	221.0
Ifosfamide	48.6	92.3
Rigosertib (ON-01910)	48.0	221.0
Pracinostat (SB939)	45.9	204.8
Ciprofibrate	45.1	4.8
Stearic acid	40.5	215.9
Troglitazone (CS-045)	40.5	1.6
Genistein	36.7	52.1
Mocetinostat (MGCD0103)	35.8	63.5
CUDC-101	33.0	87.2
Poziotinib (HM781-36B)	29.7	89.8
Fidaxomicin	27.8	149.1
Rociletinib (CO-1686, AVL-301)	24.6	98.4
Levofloxacin hydrate	24.4	174.9
Fenofibric acid	22.7	15.1
Rosiglitazone	19.2	15.8
Selumetinib (AZD6244)	16.7	153.0
Vemurafenib (PLX4032, RG7204)	15.3	33.1
Omipalisib (GSK2126458, GSK458)	15.2	60.5
Pioglitazone	15.1	12.8
Clofibric Acid	12.1	2.7
Olmutinib (HM61713, BI 1482694)	12.0	159.2
Dabrafenib (GSK2118436)	9.9	4.1
Flavopiridol (Alvocidib)	8.3	116.0
Irinotecan HCl Trihydrate	5.8	247.3
Cefoperazone sodium	4.6	57.8
Bezafibrate	4.6	142.4
Rosiglitazone maleate	3.4	62.5
Pimasertib (AS-703026)	3.3	59.8
Levofloxacin	2.7	135.9
Panobinostat (LBH589)	2.6	39.2
Trametinib (GSK1120212)	2.4	4.0
Binimetinib (MEK162, ARRY-162, ARRY-438162)	2.2	21.4
Fenofibrate	2.1	0.7

Pixantrone Maleate	2.1	117.3
Cobimetinib (GDC-0973, RG7420)	2.0	21.7
Encorafenib (LGX818)	1.8	0.8
Idarubicin HCl	1.6	193.8
Sorafenib	1.6	4.5
Raltitrexed	1.4	2.4
Dabrafenib Mesylate	1.1	2.5
YM155 (Sepantronium Bromide)	0.8	0.7
Mitomycin C	0.7	5.3
Dinaciclib (SCH727965)	0.5	0.5
Quisinostat (JNJ-26481585) 2HCl	0.3	1.1
Gemfibrozil	0.2	0.6
Rucaparib (AG-014699,PF-01367338) phosphate	0.0	217.8
Niraparib (MK-4827) tosylate	0.0	85.9
Niraparib (MK-4827)	0.0	294.7

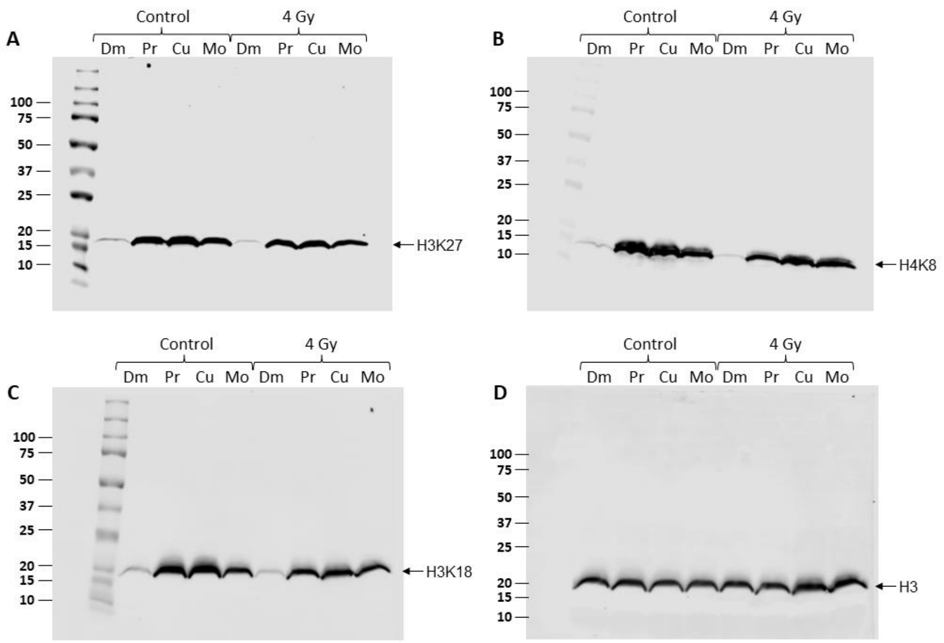

Supplementary Figure S1. Effectiveness of HDAC inhibitors in supressing histone deacetylation in FaDu cells. (**A-E**) FaDu cells were treated with DMSO, or 1 μM pracinostat (Pr), CUDC-101 (Cu) or mocetinostat (Mo), and either unirradiated (Control) or irradiated with 4 Gy X-rays and cells harvested at 2 h post-irradiation. Histones were purified by acid extraction and analysed by immunoblotting using antibodies targeted against site-specific acetylation sites on histone H3 or H4, or against unmodified histone H3. Molecular weight markers are indicated on the left-hand side of the blots and full-length blots are shown.
